# Diagnostic Performance of DNA Hypermethylation Markers in Peripheral Blood for the Detection of Colorectal Cancer: A Meta-Analysis and Systematic Review

**DOI:** 10.1371/journal.pone.0155095

**Published:** 2016-05-09

**Authors:** Bingsheng Li, Aihua Gan, Xiaolong Chen, Xinying Wang, Weifeng He, Xiaohui Zhang, Renxiang Huang, Shuzhu Zhou, Xiaoxiao Song, Angao Xu

**Affiliations:** 1 Department of Gastroenterology, Huizhou First Hospital, Huizhou, 516003, China; 2 Department of Gastroenterology, Hainan provincial people’s Hospital, Haikuo, 570100, China; 3 Department of Gastroenterology, Nanfang Hospital, Southern Medical University, Guangzhou, 510515, China; 4 Huizhou Medicine Institute, Huizhou First Hospital, Huizhou, 516003, China; 5 School of Public Health, Kunming Medical University, Kunming, 650500, China; University Hospital Llandough, UNITED KINGDOM

## Abstract

DNA hypermethylation in blood is becoming an attractive candidate marker for colorectal cancer (CRC) detection. To assess the diagnostic accuracy of blood hypermethylation markers for CRC in different clinical settings, we conducted a meta-analysis of published reports. Of 485 publications obtained in the initial literature search, 39 studies were included in the meta-analysis. Hypermethylation markers in peripheral blood showed a high degree of accuracy for the detection of CRC. The summary sensitivity was 0.62 [95% confidence interval (CI), 0.56–0.67] and specificity was 0.91 (95% CI, 0.89–0.93). Subgroup analysis showed significantly greater sensitivity for the methylated Septin 9 gene (*SEPT9*) subgroup (0.75; 95% CI, 0.67–0.81) than for the non-methylated *SEPT9* subgroup (0.58; 95% CI, 0.52–0.64). Sensitivity and specificity were not affected significantly by target gene number, CRC staging, study region, or methylation analysis method. These findings show that hypermethylation markers in blood are highly sensitive and specific for CRC detection, with methylated *SEPT9* being particularly robust. The diagnostic performance of hypermethylation markers, which have varied across different studies, can be improved by marker optimization. Future research should examine variation in diagnostic accuracy according to non-neoplastic factors.

## Introduction

Colorectal cancer (CRC) is the third most common cause of cancer-related death in the US [1]. The normal—adenoma—carcinoma sequence of colorectal carcinogenesisis is well established. The identification and management of early-stage CRC or pre-malignant lesions are highly effective interventions that substantially reduce CRC-specific mortality and morbidity. Thus, much effort is being focused on the development of early detection strategies.

Current guidelines divide CRC screening approaches into two categories: invasive and non-invasive methods [2]. Invasive methods, such as colonoscopy, remain the primary screening tools due to very good diagnostic performance, enabling the detection and removal of pre-cancerous lesions. However, invasive approaches require extensive bowel preparation, invasion of patients’ privacy, and sedation. Poor screening compliance among patients poses a challenge for CRC screening strategy development. Non-invasive screening approaches, such as fecal occult blood tests and fecal immunochemical testing (FIT), are not very effective. These methods do not detect the majority of advanced adenomas [3], and they require patient compliance in self-collecting stool samples annually for occult blood detection [4]. To date, improvements in the sensitivity and user-friendliness of feces-based tests have not increased compliance in CRC screening.

Expectations for the new generation of screening tests based on molecular biomarkers present in blood are increasing. These tests should enhance patient compliance and thereby increase early-stage CRC detection, as evidenced by the success of other screening programs, such as those based on alpha-fetoprotein for hepatocellular carcinoma and prostate-specific antigen for prostate cancer [5, 6]. In the last decade, a large body of research has revealed numerous genomic alterations associated with CRC, including ***APC*, *TP53*, *EGFR*, *BRAF***, and ***KRAS*** mutations. However, increasing evidence suggests that epigenetic changes are of similar importance as genetic alterations in the pathogenesis of CRC. Aberrant methylation of C—phosphate—G (CpG) islands in the promoter regions of genes leads to transcriptional silencing of tumor suppressor genes. Levels of free-circulating methylated DNA in the blood are increased in patients with cancer compared with healthy controls [7, 8]. DNA methylation is among the most extensively studied epigenetic processes in CRC. Analysis of specific methylated genes in CRC offers a variety of new opportunities for the development of biomarkers [9–11]. Liquid biopsies have emerged as a biomarker source. Detection of biomarkers in biological fluids offers advantages, including minimal invasiveness and easy accessibility. In particular, blood samples represent a practical source for biopsies for detection of CRC biomarkers. Hypermethylation of tumor suppressor genes in the plasma or serum of patients with CRC has been shown to hold promise as a potential methodology for the detection of this disease [12].

The detection of aberrantly methylated septin 9 (*SEPT9*) in plasma may be a valuable and non-invasive blood-based polymerase chain reaction (PCR) test, with almost 70% sensitivity and 90% specificity for detection of CRC[13–16]. The utility of methylation for CRC detection has been reported for an increasing number of genes, including ***THBD***, ***NEUROG1***, ***HIC1***, ***DAPK***, ***APC***, ***MDG1***, and ***TPEF*** [17–21]. As these epigenetic alterations occur in the early stages of tumor development, including in precancerous lesions such as adenomas, they may be useful for the diagnosis of malignant diseases.

The diagnostic performance of genetic hypermethylation biomarkers has been examined in many studies and has varied widely [12, 14, 15, 19, 21–31]. Taken together, findings suggest that such alterations could serve as efficient diagnostic markers for CRC. However, no meta-analysis with standardized inclusion criteria, data extraction, and statistical approach has been performed to provide a comprehensive overview of the accuracy and precision of these methods. The aim of this meta-analysis and review was to establish the sensitivity and specificity of hypermethylation biomarkers using data from patients with and without CRC, with histopathological findings serving as the reference standard.Patients with adenomas were not included due to the lack of sufficient high quality research reports addressing them.

## Materials and Methods

### Literature search strategy

We searched the Embase, PubMed, Cochrane Library, Ovid, Science Direct, Web of Science, and Google Scholar international databases, and four Chinese databases [Chinese National Knowledge Infrastructure, Wan Fang DATA, Chinese Biomedical Literature Database-disc, and Technology of Chongqing (VIP)] to identify relevant articles published through the 30^th^ of April 2015. The key words employed for literature retrieval were “epigenetic” or “methylation” or “hypermethylation” or “methylated” and “colorectal” or “colon” or “rectum” and “cancer” or “carcinoma” or “tumor” or “neoplasm” or “adenocarcinoma” and “serum” or “sera” or “blood” or “plasma.” To identify additional relevant articles, we scanned conference summaries and reference lists of articles identified in the initial search. We contacted authors to obtain additional information when necessary. The institutional review board of HuiZhou First Hospital waived the requirement for ethical approval.

### Inclusion and exclusion criteria

Two reviewers (B.S. Li and X.L. Chen) independently assessed all identified publications to determine their eligibility for inclusion in the study. Any disagreement was resolved by discussion until consensus was reached. Studies meeting the following criteria were included in the sample: (1) histopathological confirmation of CRC diagnoses, which is widely regarded as the gold standard; (2) peripheral blood collection before any treatment; (3) full-length article published in English or Chinese; (4) detection of hypermethylation in peripheral blood; (5) provision of sufficient data for 2 × 2table construction; and (6) inclusion of a control group consisting of patients with benign disease or healthy individuals. When two or more methods were used in a single study to detect methylation of the same target gene, data from the method with best Youden index were included in our analysis. Exclusion criteria were: (1) duplicate publication, (2) sample < 30 patients, and (3) lack of a clear cut-off value for methylation.

### Data extraction

Two reviewers (R.X. H and X.Y. Wang) independently extracted relevant data from each article using a standardized form (S1 Table). The reviewers were not blinded to the journal and author names, author affiliations, or year of publication, as this procedure has been shown to be unnecessary [32]. To resolve disagreement between reviewers, other authors assessed all discrepant items and the majority opinion was used for analysis. The following data were extracted: descriptive characteristics of the study population (age, sex, clinical stage, and sample size), study details (first author, year of publication, geographic region, target gene, and sample source), data for 2 × 2 table construction (sensitivity and specificity), and methylation analysis method.

### Quality assessment

Two reviewers (A.G. Xu and A.H. Gan) assessed the quality of each study independently using the seven-item Quality Assessment of Diagnostic Accuracy Studies (QUADAS)-2 tool [33], which has been demonstrated to be efficient for quality assessment of diagnostic accuracy studies. The quality of each item was characterized as low, high, or unclear.

### Sensitivity and specificity analyses

A bivariate meta-analysis model was employed to summarize the sensitivity, specificity, positive and negative likelihood ratios, and diagnostic odds ratio of methylation biomarker testing, and to generate a bivariate summary receiver operator characteristic (SROC) curve [34–37]. The bivariate model is considered to be a highly valid statistical model for diagnostic meta-analysis [38–41]. Because random error and clinical or methodological heterogeneity can affect study results, the *I*^2^ and *H*^2^ tests were used to assess study heterogeneity. *I*^2^ values > 50% were considered to indicate the existence of significant heterogeneity [42–44].

### Meta-regression and subgroup analyses

Meta-regression analysis was executed to determine whether diagnostic values were affected significantly by heterogeneity among studies. First, single-factor regression analysis was performed with the following variables: CRC stage (I–II/III–IV), methylated *SEPT9* (yes/no), target gene(s) (single/multiple), methylation analysis method [methylation-specific PCR (MSP)/quantitative PCR (qPCR)/other], year and region(China/other). Variables with significant regression coefficients (*P* < 0.05) were considered to be explanatory. Subsequently, we developed a multivariable regression model and used a backward stepwise algorithm to identify the variables with the most significant effects. Characteristics showing statistical significance (*P* < 0.05) were retained in the regression model.

Subgroup analyses were planned *a priori* depending on the following: identification of a highly significant effect in meta-regression analysis; number of target methylated genes examined (>3); target gene (single/multiple); region (China/other), and methylation analysis method (qPCR/MSP/other).

The publication bias of selected studies was assessed with a funnel plot and Deek’s test, which has been recommended for detection of publication bias of diagnostic test accuracy studies [45]. To detect cut-off threshold effects, the relationship between sensitivity and specificity was evaluated using the Spearman correlation coefficient. Subgroup and sensitivity analyses were also performed when necessary to dissect observed heterogeneity. All analyses were performed with Stata SE12.0 (Stata Corporation, USA) and Meta-DiSc1.4 [46] software.

## Results

### Sample characteristics

**Fig 1:**
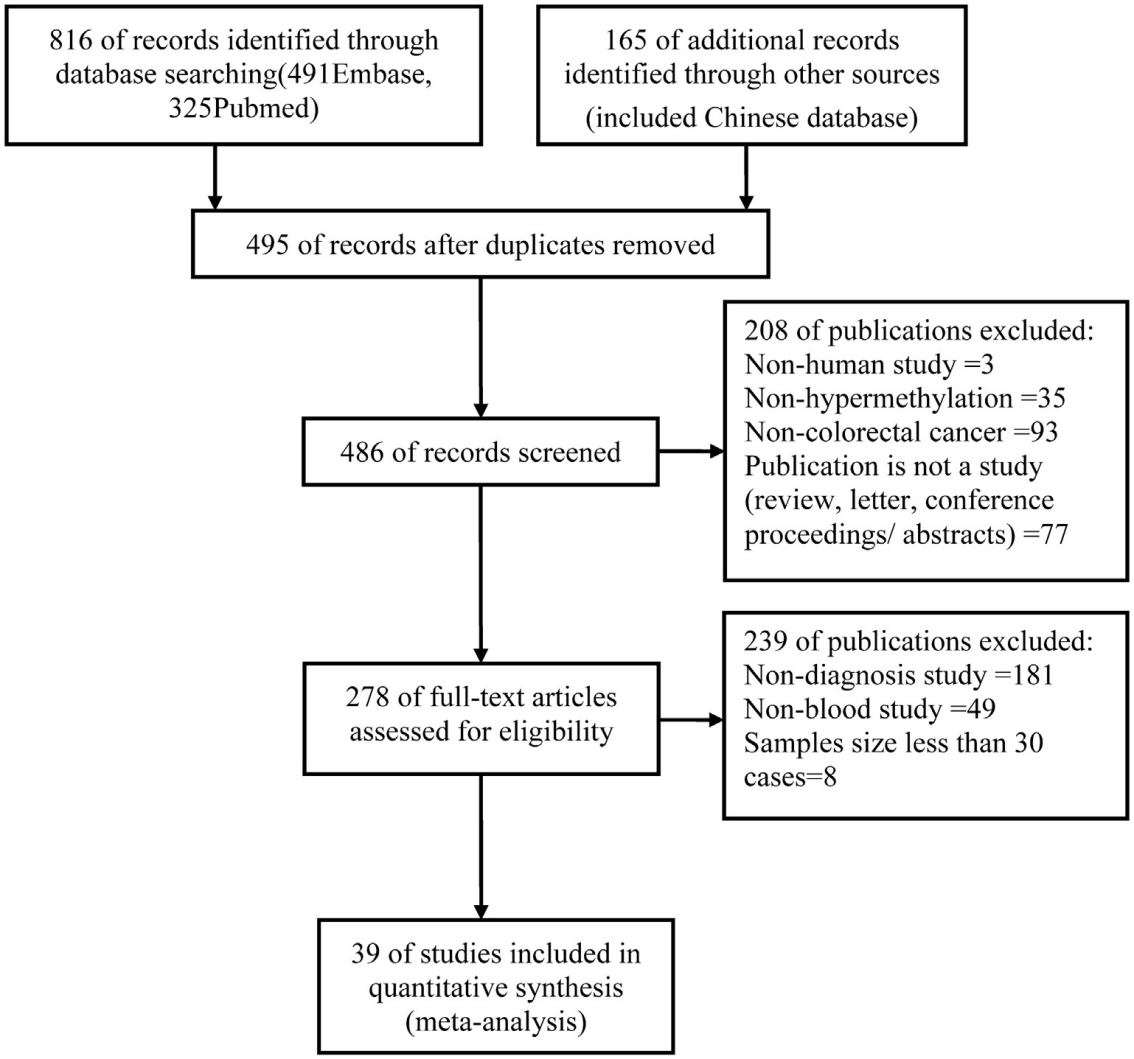
Flowchart of article selection.

The initial search returned a total of 981 articles, of which 495 duplicate publications were removed. In the next stage of assessment, 208 of the remaining 486 articles were excluded. Further evaluation led to the exclusion of 239 additional publications. After carefully reading the texts, meta-analysis was performed on the final sample of 39 studies (Fig 1), which included3853 patients with CRC and 6431 healthy controls. Studies examining advanced adenoma, the ideal target of screening, were excluded from the analysis due to the small number of publications identified and because this topic extends beyond the aim of this systematic review.

The meta-analysis sample comprised reports on the accuracy of hypermethylation markers in peripheral blood for the detection of CRC (S1 Table). Single and multiple genes were targeted, and samples were derived from serum/plasma of patients with stages I–IV CRC. The methylation analysis methods used in the analyzed studies include qPCR [14, 15, 19, 26, 30, 31, 47–60], MSP [9, 12, 17, 21, 24, 30, 31, 50–58, 61–68], and others [13–15, 17, 19, 21, 24, 26, 28, 29, 47, 59, 60, 64–71]. The reviewers recorded 198/273 (72.5%) “yes” responses and 75 (27.5%) “no/unclear” responses using the QUADAS-2 tool (S2 Table, S1 Fig). Studies were conducted on four continents: Europe (*n* = 12; 9 in Germany, 1 in Italy, 1 in Russia, and 1 in France), Australia (*n* = 2), Asia (*n* = 22; 16 in China, 2 in South Korea, and 4 in Japan), and North America (*n* = 5, all in the USA). Of the16 studies carried out in China, 6 were Chinese-language publications [21, 47, 53, 54, 57, 68].

### Summary performance estimates

**Fig 2:**
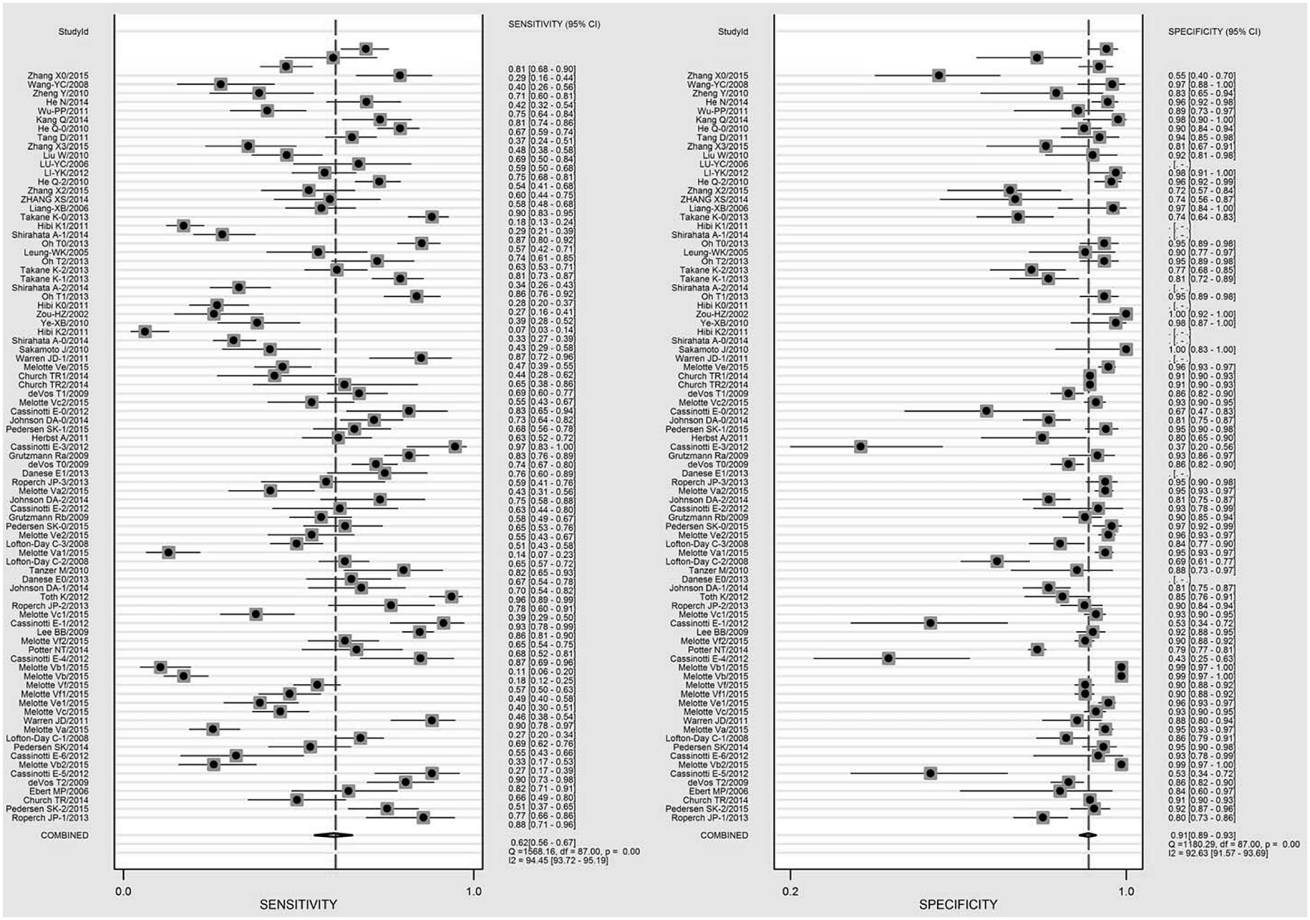
Forest plots of summary performance estimates.

The pooled sensitivity and specificity estimates were 0.62 [95% confidence interval (CI), 0.56–0.67; *I*^2^ = 94.5%, *H*^2^ = 18.0] and 0.91 (95% CI, 0.89–0.93; *I*^2^ = 92.6%, *H*^2^ = 13.5), respectively (Fig 2). The area under the curve was 0.87 (95% CI, 0.84–0.90). Pooled and hierarchical SROC curves are shown in S2 Fig. The effects of diagnostic threshold and publication bias were not significant (*t* = -0.96, *P* = 0.34 and *t* = -0.47, *P* = 0.68, respectively). The results of the analysis of pooled data from all of the analyzed studies are shown in Table 1 and S3 Fig.

**Table 1:** Summary performance estimates.

Parameter	Estimate	95% CI	*I*^2^ (95% CI)	*H*^*2*^ (95% CI)	*tau-square*
Sensitivity	0.62	0.56, 0.67	94.5 (93.7,95.2)	18.0 (15.9,20.8)	-
Specificity	0.91	0.89, 0.93	92.6 (91.6,93.7)	13.5 (11.9,15.9)	-
PLR	6.9	5.8, 8.2	84.0 (83.2,88.7)	6.3 (6.0,8.8)	0.33
NLR	0.42	0.37, 0.48	94.0 (93.2,94.8)	16.7 (14.7,19.2)	0.67
DOR	16	13, 20	-		0.54

CI, confidence interval; PLR, positive likelihood ratio; NLR, negative likelihood ratio DOR, diagnostic odds ratio.

### Meta-regression and subgroup analyses

Single-factor regression analysis showed that only the methylated *SEPT9* variable was explanatory. Multivariable regression showed that this variable had most statistically significant difference. Geographic region and methylation analysis method contributed significantly to heterogeneity among studies; staging did not affect pooled performance (Table 2).

**Table 2:** Meta-regression analysis results.

Parameter category	χ^2^	*P*	*I*^2^	*I*^2^ low	*I*^2^ high
Method	3.89	0.14	49	0	100
Target gene	4.12	0.13	51	0	100
CRC stage	1.48	0.48	0	0	100
Region	0.06	0.97	0	0	100
mSEPT9	7.96	0.02	75	45	100
Year	1.20	0.55	0	0	100

CRC, colorectal cancer; mSEPT9, aberrantly methylated *SEPT9*.

The results of subgroup analyses are provided in Table 3. Sensitivity was significantly higher in the methylated *SEPT9* subgroup than in the non-methylated *SEPT9* subgroup, but neither demonstrated significant specificity. No significant difference among other subgroups was observed. The PRISMA 2009 checklist is provided as S2 File.

**Table 3:** Subgroup analysis results.

Parameter	Sen (95% CI)	*I*^2^ (95% CI)	Spe (95% CI)	*I*^2^ (95% CI)
Total	0.62 (0.56, 0.67)	94.5 (93.7,95.2)	0.91 (0.89, 0.93)	92.6 (91.6,93.7)
Single targeted gene	0.60 (0.53, 0.65)	94.3 (93.5,95.1)	0.91 (0.89, 0.93)	93.1 (92.1,94.2)
Multiple targeted gene	0.74 (0.65, 0.82)	92.0 (88.5,95.4)	0.87 (0.81, 0.92)	91.5 (87.8,95.2)
mSEPT9	0.75 (0.67, 0.81)	82.4 (74.8,89.9)	0.89 (0.86, 0.92)	94.8 (93.2,96.4)
Non- mSEPT9	0.58 (0.52, 0.64)	94.7 (93.9,95.4)	0.91 (0.89, 0.93)	91.2 (90.5,93.2)
q-PCRmethod	0.69 (0.58, 0.79)	96.1 (94.9,97.2)	0.88 (0.83, 0.91)	76.0 (64.4,87.6)
MSP method	0.54 (0.46, 0.61)	95.0 (94.1,95.8)	0.93 (0.91, 0.95)	90.2 (90.5,93.2)
Other method	0.72 (0.64, 0.79)	85.3 (79.9,90.5)	0.85 (0.78, 0.90)	94.7 (93.2,96.1)
China region	0.62 (0.55, 0.69)	88.3 (84.0,92.5)	0.91 (0.89, 0.93)	87.2 (82.4,91.9)
Other region	0.60 (0.53, 0.67)	95.2 (94.5,95.9)	0.91 (0.86, 0.94)	93.7 (92.4,94.5)

Sen, sensitivity; CI, confidence interval; Spe, specificity; mSEPT9, aberrantly methylated septin 9; PCR, polymerase chain reaction; MSP, methylation-specific PCR.

## Discussion

Promoter hypermethylation analysis of blood DNA has the potential to serve as a non-invasive screening method for the identification of specific biomarkers, enabling early detection of CRC. This approach holds promise for increased accuracy, safety, affordability, and patient compliance [16, 72].

However, detection of early-stage and pre-cancerous lesions is hampered by the uncertain performance of non-invasive screening tests [72]. In this meta-analysis, we evaluated the diagnostic and clinical value of DNA hypermethylation as a serological marker for the diagnosis of CRC. The pooled sensitivity of tests detecting aberrant methylation in blood was significantly lower than that reported for FIT(0.79; 95% CI,0.69–0.86) [73] and stool DNA methylation testing (0.75–0.78; 95% CI,0.69–0.82) [74, 75], but specificity did not differ significantly (0.94, 95% CI 0.92–0.95 [73, 76] and 0.90–0.91,95% CI 0.86–0.96, respectively). Sensitivity and specificity values for methylated *SEPT9* and multiple target gene subgroups were consistent with those for FIT and stool DNA methylation testing, with the advantage of better acceptability and compliance of serological testing for the purpose of CRC screening.

We observed a large degree of heterogeneity among studies for all blood DNA methylation tests used in CRC screening, due primarily to study region and methylation analysis method. The lack of a significant effect of CRC stage may be due to the lack of CRC staging in the majority of studies and/or the inclusion of few patients with stages I–II CRC. More research on early-stage CRC is expected in the future.

Theoretically, the use of multiple biomarkers for cancer screening is more accurate than the use of a single marker. Although polygenetic testing showed greater sensitivity in this analysis, neither sensitivity nor specificity differed significantly between studies targeting single and multiple genes. These results may be explained by the following: (1) the mechanisms underlying CpG methylation in CRC remain unclear; (2) no highly accurate CRC-specific methylated target gene has been identified due to the heterogeneity of colon cancer; (3) diverse methods of gene methylation testing may produce different results; and (4) the sample of polygene studies was far smaller than that of single-gene studies, and the diagnostic accuracy of the former was not superior to that of the latter.

Consistent with the results of the present review, some independent case-control studies have suggested that methylated *SEPT9* in plasma can be used to identify individuals with CRC with 0.52–0.72 sensitivity and 0.90–0.95specificity [14, 16]. In a recent prospective study of 7941 cases, Church et al. [71] obtained sensitivity and specificity estimates of 0.48 (95% CI, 0.32–0.64) and 0.92 (95% CI, 0.90–0.93), respectively, for methylated *SEPT9*. In the present analysis, methylated *SEPT9* detection showed significantly greater sensitivity than methods that did not use this gene, but no advantage in terms of specificity. Methylated *SEPT9* has been shown to be a good marker for CRC screening, but its potential has not been explored fully due to the high degree of heterogeneity, which affects its clinical availability.

Each DNA methylation analysis technique has inherent advantages and disadvantages, and procedures vary substantially [77]. These factors have prevented the formulation of unified standards and have made cross-validation studies problematic. No single technique or approach is optimal because none combines quantitative accuracy, sensitive detection, high local or global informational content, sample source compatibility, and procedure automation. Thus, technique selection affects laboratory results and may lead to heterogeneity. In the present study, the examination of findings according to methylation analysis method led to no significant reduction in heterogeneity and no difference insensitivity or specificity. Thus, further improvement in methylation detection methodologies is required.

Epigenetics results from the interaction between genetic material and environmental factors. The level of DNA methylation *in vivo* is regulated by genetic, lifestyle, and dietary factors [78, 79]. In the present analysis, the accuracy of studies conducted in China and in other regions did not differ, but studies conducted in China showed a high degree of heterogeneity. This result may be due to lifestyle (smoking and alcohol consumption), dietary (folate and tea intake), and environmental exposure factors.

Diagnostic test accuracy meta-analyses have different characteristics and more heterogeneity than therapeutic/interventional meta-analyses due to a variety of reasons [35, 80], including the use of non-randomized trials, natural variation in sensitivity and specificity across positivity thresholds (cut-offs), and differences in design, conduct, protocols, and reference standards. Assessment of the potential for publication and related bias was more complicated than for intervention reviews, and investigation of the influence of publication bias in terms of small study-effects is challenging. The bivariate and HSROC models used in this review are currently the most statistically rigorous methods for diagnostic test accuracy meta-analysis and are recommended by authoritative bodies such as the Cochrane Collaboration and the AHRQ [34–37]. In addition, we used a variety of statistical methods to find and analyze possible sources of heterogeneity.

The inability to control for sources of heterogeneity effectively in this study may be due to: 1) lack of clarity regarding the causal relationship between methylation and CRC; 2) substantial heterogeneity of current methylation markers in tumors; 3) effects of many non—tumor-related factors, such as genovariation, aging, sex, hormone levels, lifestyle factors (smoking and alcohol consumption), race, inflammation, diet, and environmental factors, on methylation [78, 79]; 4) diversity and limitations of existing methylation detection methods, which hamper procedural standardization; 5) lack of knowledge about the mechanisms underlying publication bias in diagnostic test accuracy studies resulting in the possibility of weak power for statistical methods for detection of selective publication bias [45], and 6) examination of the majority of diagnostic tests in case-control studies, while prospective randomized controlled trials are rare.

This meta-analysis has several limitations. Most publications included in the analysis reported on case-control studies with small samples. In addition, the effects of language selection bias and literature type cannot be ignored in any meta-analysis. Finally, the included studies did not account for the effects of aging, sex, lifestyle, diet, and methodology on their findings.

In conclusion, the use of serological methylated markers should be the approach of choice for CRC screening due to greater diagnostic performance, convenience, and compliance in comparison with non-serological methods. Methylated *SEPT9* showed relatively high pooled sensitivity, which was affected by many factors, but not by CRC staging. Much further work to reduce the high level of heterogeneity is needed before serological methylated markers are applied widely for CRC screening in clinical practice. Future clinical diagnostic studies of methylation in blood should consider the impacts of marker optimization and non-neoplastic factors (e.g., aging, sex, lifestyle, diet, methodology) on diagnostic accuracy. In addition, more studies of early CRC screening are expected. As serological methylation detection is a relatively new CRC screening method, improvements in accuracy can be expected as the diagnostic technology evolves.

## Supporting Information

S1 Fig Graphical display of quality assessment results. (TIF)

S2 Fig Pooled and hierarchical summary receiver operating characteristic curves. (TIF)

S3 Fig Deek’s funnel plot asymmetry test. (TIF)

S1 File Meta-analysis-on-genetic-association-studies-form. (DOC)

S2 File PRISMA 2009 checklist. (DOC)

S3 File PRISMA 2009 flow diagram. (DOC)

S1 Table Characteristics of the 39 included studies. (DOCX)

S2 Table Quality of included studies. (DOC)
