# Supplementary figures and images for: Diagnostic Performance of DNA Hypermethylation Markers in Peripheral Blood for the Detection of Colorectal Cancer: A Meta-Analysis and Systematic Review
Source: PLoS One. 2016 May 9;11(5):e0155095. doi: 10.1371/journal.pone.0155095 (PMC4861294; doi:10.1371/journal.pone.0155095)

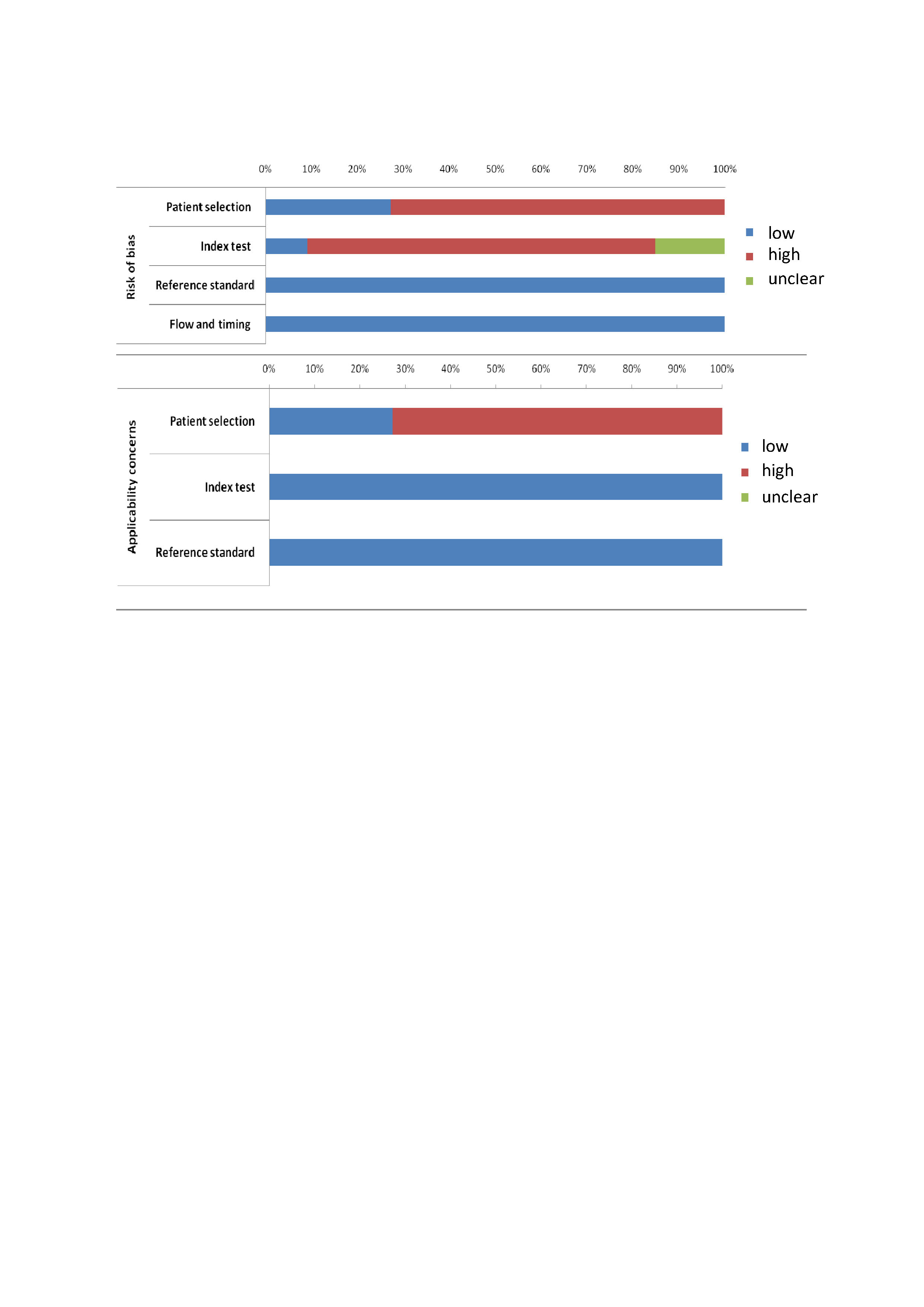

Supplement: S1 Fig — (TIF) [file pone.0155095.s001.tif]

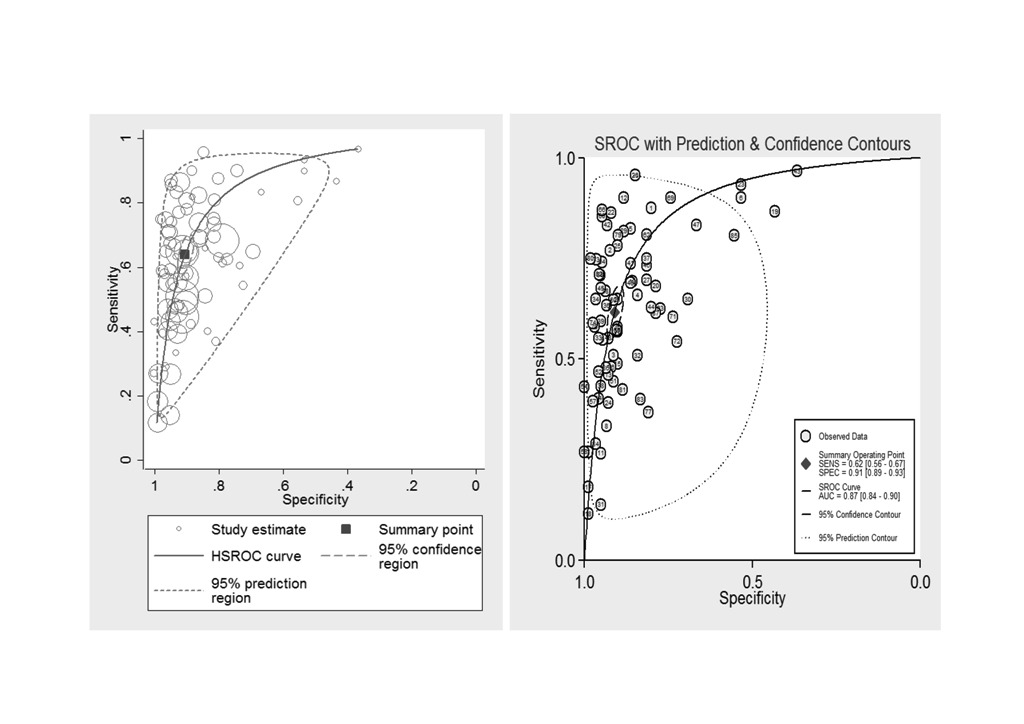

Supplement: S2 Fig — (TIF) [file pone.0155095.s002.tif]

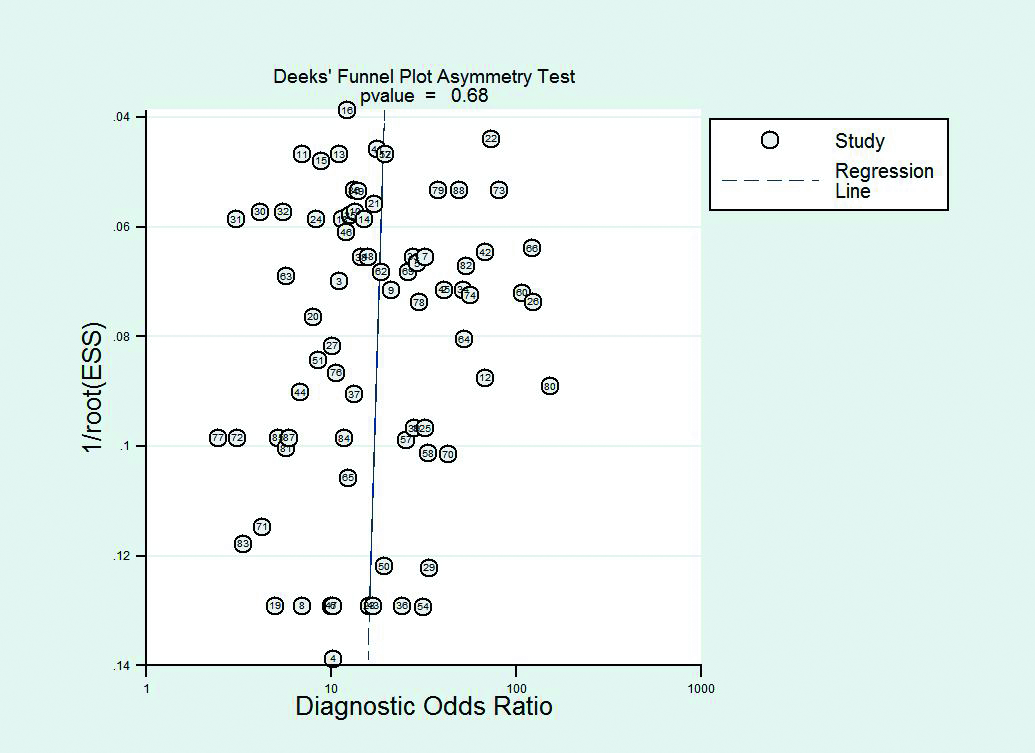

Supplement: S3 Fig — (TIF) [file pone.0155095.s003.tif]
