# Supplementary material for: Diagnostic Performance of DNA Hypermethylation Markers in Peripheral Blood for the Detection of Colorectal Cancer: A Meta-Analysis and Systematic Review
Source: PLoS One. 2016 May 9;11(5):e0155095. doi: 10.1371/journal.pone.0155095 (PMC4861294; doi:10.1371/journal.pone.0155095)
Supplement: S3 File — (DOC) [file pone.0155095.s006.doc]

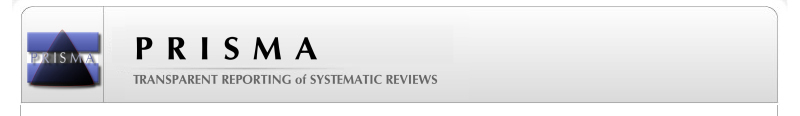
**PRISMA 2009 Flow Diagram**

**Screening**

**Included**

**Eligibility**

**Identification**

Records identified through database searching
(n = 981 )

Additional records identified through other sources
(n = 0 )

Records after duplicates removed
(n = 495 )

Records screened
(n =486 )

Records excluded
(n = 208 )

Full-text articles assessed for eligibility
(n = 278 )

Full-text articles excluded, with reasons
(n =239 )

Studies included in qualitative synthesis
(n =39 )

Studies included in quantitative synthesis (meta-analysis)
(n = 39 )
