# Supplementary material for: Diagnostic Performance of DNA Hypermethylation Markers in Peripheral Blood for the Detection of Colorectal Cancer: A Meta-Analysis and Systematic Review
Source: PLoS One. 2016 May 9;11(5):e0155095. doi: 10.1371/journal.pone.0155095 (PMC4861294; doi:10.1371/journal.pone.0155095)
Supplement: S1 Table — (DOCX) [file pone.0155095.s007.docx]

**S1Table.**Characteristics of the 39included studies

| **Study** | **CRC stage** | **Control** | **Population(case/control)** | **Age (years)** | **Sex (M/F)** | **Region** | **Target methylated gene** | **Plasma or serum** | **Blinding** | **Analytical method** | **Sensitivity^a^** | **Specificity** | **TP** | **FN** | **FP** | **TN** |
| --- | --- | --- | --- | --- | --- | --- | --- | --- | --- | --- | --- | --- | --- | --- | --- | --- |
| Pedersen et al.2015(61) | I–IV | HP | 218 (74/144) | 32–86 | 113/105 | Australia | BCAT1 | P | Yes | qMSP | 64.9% | 96.5% | 48 | 26 | 5 | 139 |
|  |  |  |  |  |  |  | IKZF1 |  |  |  | 67.6% | 95.1% | 50 | 24 | 7 | 137 |
|  |  |  |  |  |  |  | jointed |  |  |  | 77.0% | 92.4% | 57 | 17 | 11 | 133 |
| Zhang et al.2015(62) | I–IV | HP | 104 (/57/47) | - | - | China | GATA5 | P | Unclear | MSP | 61.40% | 78.72% | 35 | 22 | 10 | 37 |
|  |  |  |  |  |  |  | SFRP2 |  |  |  | 54.39% | 72.34% | 31 | 26 | 13 | 34 |
|  |  |  |  |  |  |  | ITGA4 |  |  |  | 36.84% | 80.85% | 21 | 36 | 9 | 38 |
|  |  |  |  |  |  |  | jointed |  |  |  | 80.7% | 55.32% | 46 | 11 | 21 | 26 |
| Melotteet al.2015(63) | I–IV | HP | 596 (154/442) | - | - | Germany | NDRG4 | P | Unclear | MSP | 27.0% | 95.0% | 41 | 113 | 22 | 420 |
|  | I, II |  | 529 (87/442) | - | - |  |  |  |  |  | 13.8% | 95.0% | 12 | 75 | 22 | 420 |
|  | III, IV |  | 509 (67/442) | - | - |  |  |  |  |  | 43.28% | 95.0% | 29 | 38 | 22 | 420 |
|  | I–IV |  | 598 (154/444) |  |  |  | GATA5 |  |  |  | 18.0% | 99.0% | 28 | 126 | 5 | 439 |
|  | I, II |  | 531 (87/444) |  |  |  |  |  |  |  | 11.49% | 99.0% | 10 | 77 | 5 | 439 |
|  | III, IV |  | 511 (67/444) | - | - |  |  |  |  |  | 26.87% | 99.0% | 18 | 49 | 5 | 439 |
|  | I–IV |  | 598 (154/444) |  |  |  | FOXE1 |  |  |  | 46.0% | 93.0% | 71 | 83 | 32 | 412 |
|  | I, II |  | 531 (87/444) |  |  |  |  |  |  |  | 39.08% | 93.0% | 34 | 53 | 32 | 412 |
|  | III, IV |  | 511 (67/444) |  |  |  |  |  |  |  | 55.22% | 93.0% | 37 | 30 | 32 | 412 |
|  | I–IV |  | 598 (154/444) |  |  |  | SYNE1 |  |  |  | 47.0% | 96.0% | 72 | 82 | 19 | 425 |
|  | I, II |  | 531 (87/444) |  |  |  |  |  |  |  | 40.2% | 96.0% | 35 | 52 | 19 | 425 |
|  | III, IV |  | 511 (67/444) |  |  |  |  |  |  |  | 55.22% | 96.0% | 37 | 30 | 19 | 425 |
|  | I–IV |  | 904 (220/684) |  |  |  | FOXE1/ SYNE1 |  |  |  | 56.81% | 89.14% | 125 | 95 | 67 | 617 |
|  | I, II |  | 813 (129/684) |  |  |  |  |  |  |  | 48.84% | 89.14% | 63 | 66 | 67 | 617 |
|  | III, IV |  | 775 (91/684) |  |  |  |  |  |  |  | 64.84% | 89.14% | 59 | 32 | 67 | 617 |
| Potter et al. 2014(69) | I–III | Non-CRC | 1544 (44/1500) | ≥50 | 819/725 | Germany | SEPT9 | P | Unclear | Epi proColon assay | 64.0% | 80.0% | 30 | 14 | 318 | 1182 |
| Kang et al.2014(47) | I–IV | Non-CRC | 132 (80/52) |  | - | China | SEPT9 | P | Yes | qPCR | 75.0% | 98.1% | 60 | 20 | 1 | 51 |
| Johnson et al.2014(70) | I–IV | Non-CRC | 301 (101/200) | ≥50 | 146/155 | USA | SEPT9 | P | Unclear | Epi proColon assay | 73.3% | 81.5% | 74 | 27 | 37 | 163 |
|  | I, II |  | 46/200 |  | - |  |  |  |  |  | 69.6% | 81.5% | 32 | 14 | 37 | 163 |
|  | III, IV |  | 36/200 |  | - |  |  |  |  |  | 75.0% | 81.5% | 27 | 9 | 37 | 163 |
| Shirahataet al.2014(48) | I–IV | - | 242 | - | 140/102 | Japan | VIM | S | Unclear | qPCR | 32.6% | - | 78 | 161 | - | - |
|  | I, II |  | 109 |  |  |  |  |  |  |  | 29.4% | - | 32 | 77 | - | - |
|  | III, IV |  | 123 |  |  |  |  |  |  |  | 34.1% | - | 42 | 81 | - | - |
| Zhang et al.2014(64) | I–IV | HP | 77 (43/34) | - | - | China | GATA5 | P | Unclear | MSP | 60.7% | 73.5% | 26 | 17 | 9 | 25 |
| He et al.2014(49) | I–IV | HP | 281 (76/205) | - | - | China | SEPT9 | S | Unclear | qPCR | 71.1% | 95.6% | 54 | 22 | 9 | 196 |
| Pedersen et al.2014(65) | I–IV | Non-CRC | 220 (73/147) | - | - | Australia | CAHM (RNA) | P | Unclear | qMSP | 55.0% | 94.6% | 40 | 33 | 8 | 139 |
| Church et al.2014(71) | I–IV | Non-CRC | 1510 (53/1457) | ≥50 | - | USA &Germany | SEPT9 | P | Unclear | Epi proColon assay | 50.9% | 91.4% | 27 | 26 | 126 | 1331 |
|  | I, II |  | 36/1457 |  |  |  |  | P | Unclear |  | 44.4% | 91.4% | 16 | 20 | 126 | 1331 |
|  | III, IV |  | 17/1457 | - | - |  |  | P | Unclear |  | 64.7% | 91.4% | 11 | 6 | 126 | 1331 |
| Oh et al.2013(50) | I–IV | HP | 256 (131/115) | 33–84 | 132/123 | South Korea | SDC2 | S | Unclear | qMSP | 87.0% | 95.2% | 114 | 17 | 6 | 109 |
|  | I, II |  | 83/115 |  |  |  |  | S | Unclear |  | 85.5% | 95.2% | 71 | 12 | 6 | 109 |
|  | III, IV |  | 58/115 |  |  |  |  | S | Unclear |  | 74.1% | 95.2% | 43 | 15 | 6 | 109 |
| Daneseet al.2013(9) | Early,  advanced | - | 67 | - | 41/22 | Italy | PCDH10 | P | No | MSP | 66.7% | - | 42 | 21 | - | - |
|  | Early | - | 38 | - | - |  |  | P | Unclear |  | 76.3% | - | 29 | 9 | - | - |
|  | Advanced | - | 25 | - | - |  |  | P | Unclear |  | 56.0% | - | 14 | 11 | - | - |
| Takaneet al.2013(66) | I–IV | Non-CRC | 217 (120/97) | - | - | Japan | Joint | P | Unclear | MSP | 90% | 64% | 108 | 12 | 25 | 72 |
|  |  |  |  | - | - |  | PPP1R3C | P | Unclear |  | 81% | 81% | 97 | 23 | 18 | 79 |
|  |  |  |  | - | - |  | EFHD1 | P | Unclear |  | 63% | 78% | 75 | 45 | 21 | 72 |
| Roperchet al.2013(51) | I–IV | Non-CRC | 32/161 | - | 107/86 | France | NPY | S | Unclear | QM-MSP | 87% | 80% | 28 | 4 | 32 | 129 |
|  |  |  |  |  |  |  | PENK | S | Unclear |  | 78% | 90% | 25 | 7 | 16 | 145 |
|  |  |  |  | - | - |  | WIF1 | S | Unclear |  | 59% | 95% | 19 | 13 | 8 | 153 |
| Li et al.2012(68) | - | Non-CRC | 197 (117/80) | - | - | China | SFRP2 | P | Unclear | MSP | 59.0% | 97.5% | 69 | 48 | 2 | 78 |
| Tothet al.2012(59) |  | HP | 184 (92/92) |  | 103/84 | Germany | SEPT9 | P | Unclear | qPCR | 95.7% | 84.8% | 88 | 4 | 14 | 78 |
| Cassinotti et al.2012(17) | I (11), II (19) | HP | 60 (30/30) | 40–85 | - | USA | RASSF1A | P | Unclear | Microarray-mediated methylation assay | 93% | 53% | 28 | 2 | 14 | 16 |
|  |  |  |  |  |  |  | HIC1 | P | Unclear |  | 63% | 93% | 19 | 11 | 2 | 28 |
|  |  |  |  |  |  |  | CYCD2 | P | Unclear |  | 97% | 37% | 29 | 1 | 19 | 11 |
|  |  |  |  |  |  |  | PAX5 | P | Unclear |  | 87% | 43% | 26 | 4 | 17 | 13 |
|  |  |  |  |  |  |  | RB1 | P | Unclear |  | 90% | 53% | 27 | 3 | 14 | 16 |
|  |  |  |  |  |  |  | SRBC | P | Unclear |  | 33% | 93% | 10 | 20 | 2 | 28 |
|  |  |  |  |  |  |  | Joint | P | Unclear |  | 83.7% | 67.9% | 25 | 5 | 10 | 20 |
| Tang et al.2011(52) | I－IV | Non-CRC | 169/63 | - | - | China | SFRP2 | S | Unclear | MSP | 66.9% | 93.7% | 113 | 56 | 4 | 59 |
| Warrenet al.2011(60) | I–IV | Non-CRC | 144 (50/94) | - | - | USA &Russia | SEPT9 | P | Unclear | qPCR | 90% | 88.3% | 45 | 5 | 11 | 83 |
|  | I, II |  | 38 |  |  |  |  | P | Unclear |  | 86.8% |  | 33 | 5 | - | - |
| Herbstet al.2011(19) | I, II | HP | 97/45 | - | 61/81 | Germany | NEUROG1 | S | Unclear | MethyLight | 62.9% | 80% | 61 | 36 | 9 | 36 |
| Hibiet al.2011(31) | I–IV | - | 215 | 27–89 | 125/90 | Japan | TFPI2 | S | No | qMSP | 18% | - | 39 | 176 | - | - |
|  | I, II | - | 100 | - | - |  |  | S | No |  | 7% |  | 7 | 93 | - | - |
|  | III, IV | - | 115 | -- | - |  |  | S | No |  | 27.8% |  | 32 | 83 | - | - |
| Wuet al.2011(30) | I–IV | Non-CRC | 85/35 | - | 48/37 | China | DLC1 | S | Unclear | MSP | 42.4% | 88.6% | 36 | 49 | 4 | 31 |
| Liu et al.2010(29) | I–IV | HP | 100/50 | - | - | China | AKAP12 | P | Unclear | MS-HRM | 48% | 92% | 48 | 52 | 4 | 46 |
| Zheng et al.2010(53) | - | Non-CRC/HP | 45/30 |  | 25/20 | China | RUNX3 | S | Unclear | MSP | 40% | 83.3% | 18 | 27 | 5 | 25 |
| Tanzeret al.2010(28) | I–IV | HP | 33/34 | 52–72 | - | Germany | SEPT9/ALX4 | P | Unclear | Heavy MethyLight  qPCR | 81.8% | 86.7% | 27 | 6 | 4 | 30 |
| Ye et al.2010(54) | - | Non-CRC | 71/40 | - | - | China | DLEC1 | S | Unclear | MSP | 39.4% | 97.5% | 28 | 43 | 1 | 39 |
| He et al.2010(67) | I–IV | HP | 182/170 | 58（-） | 121/61 | China | ALX4 | P | Unclear | multiplex MethyLight | 47.8% | 93.5% | 87 | 95 | 11 | 159 |
|  |  |  |  |  |  |  | SEPT9 | P | Unclear |  | 74.7% | 96.4% | 136 | 46 | 6 | 164 |
|  |  |  |  |  |  |  | TMEFF2 | P | Unclear |  | 70.9% | 95.3% | 129 | 53 | 8 | 162 |
|  |  |  |  |  |  |  | Joint | P | Unclear |  | 80.8% | 90% | 147 | 35 | 17 | 153 |
| Sakamoto et al.2010(48) | I–IV | Non-CRC | 51/20 | - | - | Japan | P16 | S | Unclear | IP-MSP | 43.1% | 100% | 22 | 29 | 0 | 20 |
| deVoset al.2009(14) | I–IV | Non-CRC | 187/327 | 37–87 | 115/72 | Germany | SEPT9 | P | Unclear | qPCR | 73.8% | 86.2% | 138 | 49 | 45 | 282 |
|  | I, II |  | 119/327 |  |  |  |  | P | Unclear |  | 68.9% | 86.2% | 82 | 37 | 45 | 282 |
|  | III, IV |  | 68/327 |  |  |  |  | P | Unclear |  | 82.4% | 86.2% | 56 | 12 | 45 | 282 |
| Lee et al.2009(12) | I, II | HP | 243/276 | 61 | 139/104 | South Korea | APC/MGMT /RASSF2A /Wif-1 | P | Unclear | MSP | 86.5% | 92.1% | 210 | 33 | 22 | 254 |
| Grutzmannet al.2009(15) | I–IV | HP | 252/102 | - | 144/108 | Germany | SEPT9 | P | Unclear | qPCR | 83.3% | 93.1% | 120 | 24 | 7 | 95 |
|  |  |  | 126/183 |  | 76/50 |  |  | P | Unclear |  | 57.9% | 90.2% | 73 | 53 | 18 | 165 |
| Lofton-Day et al.2008(13) | I–IV | Non-CRC/HP | 179/133 | - | - | Germany | SEPT9 | P | Unclear | HM-qPCR | 69% | 14% | 124 | 55 | 19 | 114 |
|  |  |  |  |  |  |  | NGFR | P | Unclear |  | 65% | 31% | 116 | 63 | 41 | 92 |
|  |  |  |  |  |  |  | TMEFF2 | P | Unclear |  | 51% | 16% | 91 | 88 | 21 | 112 |
| Wanget al.2008(56) | I–IV | Non-CRC | 45/60 | - | - | China | RASSF1A | S | Unclear | MSP | 28.9% | 96.7% | 13 | 32 | 2 | 58 |
| Zhang et al.2006(57) | - | Non-CRC | 100/32 |  | - | China | P16 | S | Unclear | MSP | 58% | 92.4% | 58 | 42 | 1 | 31 |
| Luet al.2006(21) | I–IV | - | 32 | 22-68 | 23/9 | China | TPEF | P | No | MSP | 68.8% |  | 22 | 10 | - | - |
| Ebert et al.2006(26) | I–IV | Non-CRC | 30/30 | - | - | USA | ALX4 | S | Unclear | MethyLight | 90% | 53.3% | 27 | 14 | 3 | 16 |
| Leunget al.2005(58) | I–IV | H-P | 49/41 | - | - | China | APC/hMLH1/HLTF | S | Yes | MSP | 57.1% | 90.2% | 28 | 21 | 4 | 37 |
| Zou2002(24) | A–D(Dukes’) | Non-CRC | 52/44 | - | - | China | P16 | P | Unclear | MSP | 26.9% | 100% | 14 | 38 | 0 | 44 |

^a^Sensitivity was defined as the fraction of confirmed CRC cases in which methylation of a marker was found in serum or plasma, regardless of whether methylation of that marker was present in the associated tumor or whether the associated tumor was analyzed for the presence of the marker.

CRC, colorectal cancer; TP, true positive; FN, false negative; FP, false positive; TN, true negative; HP, healthy population; Non-CRC, non colorectal cancer.
