# Supplementary material for: Diagnostic Performance of DNA Hypermethylation Markers in Peripheral Blood for the Detection of Colorectal Cancer: A Meta-Analysis and Systematic Review
Source: PLoS One. 2016 May 9;11(5):e0155095. doi: 10.1371/journal.pone.0155095 (PMC4861294; doi:10.1371/journal.pone.0155095)
Supplement: S2 Table — (DOC) [file pone.0155095.s008.doc]

**Table S2.** Summary of quality factors of included studies.

| **Study** | **Risk of bias** | | | | **Applicability concerns** | | |
| --- | --- | --- | --- | --- | --- | --- | --- |
|  | **Patient selection** | **Index test** | **Reference standard** | **Flow and timing** | **Patient selection** | **Index test** | **Reference standard** |
| Pedersen et al. 2015^[61]^ | Yes | Yes | Yes | Yes | Yes | Yes | Yes |
| Zhang et al. 2015^[62]^ | Yes | No | Yes | Yes | Yes | Yes | Yes |
| Melotte et al. 2015^[63]^ | Yes | Yes | Yes | Yes | Yes | Yes | Yes |
| Potter et al. 2014^[69]^ | Yes | Yes | Yes | Yes | Yes | Yes | Yes |
| Kang et al.2014(47) | Yes | Yes | Yes | Yes | Yes | Yes | Yes |
| Johnson et al. 2014^[70]^ | Yes | No | Yes | Yes | Yes | Yes | Yes |
| Shirahata et al. 2014^[48]^ | No | No | Yes | Yes | Yes | Yes | Yes |
| Zhang et al. 2014^[64]^ | No | No | Yes | Yes | Yes | Yes | Yes |
| Na et al. 2014^[49]^ | Yes | No | Yes | Yes | Yes | Yes | Yes |
| Pedersen et al. 2014^[65]^ | No | No | Yes | Yes | Yes | Yes | Yes |
| Church et al. 2014^[71]^ | Yes | Yes | Yes | Yes | Yes | Yes | Yes |
| Oh et al. 2013^[50]^ | No | No | Yes | Yes | No | Yes | Yes |
| Danese et al. 2013^[9]^ | Yes | No | Yes | Yes | Yes | Yes | Yes |
| Takane et al. 2013^[66]^ | No | No | Yes | Yes | No | Yes | Yes |
| Roperch et al. 2013^[51]^ | Yes | No | Yes | Yes | Yes | Yes | Yes |
| Li et al. 2012^[68]^ | No | Unclear | Yes | Yes | No | Yes | Yes |
| Toth et al. 2012^[59]^ | No | Unclear | Yes | Yes | No | Yes | Yes |
| Cassinotti et al. 2012^[17]^ | No | No | Yes | Yes | No | Yes | Yes |
| Tang et al. 2011^[52]^ | Yes | No | Yes | Yes | Yes | Yes | Yes |
| Warren et al. 2011^[60]^ | Yes | Yes | Yes | Yes | Yes | Yes | Yes |
| Herbst et al. 2011^[19]^ | No | No | Yes | Yes | No | Yes | Yes |
| Hibi et al. 2011^[31]^ | No | No | Yes | Yes | No | Yes | Yes |
| Wu et al. 2011^[30]^ | No | Unclear | Yes | Yes | No | Yes | Yes |
| Liu et al. 2010^[27]^ | No | Unclear | Yes | Yes | No | Yes | Yes |
| Zheng et al. 2010^[53]^ | No | Unclear | Yes | Yes | No | Yes | Yes |
| Tanzer et al. 2010^[28]^ | Yes | No | Yes | Yes | Yes | Yes | Yes |
| Ye et al. 2010^[54]^ | No | No | Yes | Yes | No | Yes | Yes |
| He et al. 2010^[67]^ | No | No | Yes | Yes | No | Yes | Yes |
| Sakamoto et al. 2010^[55]^ | No | No | Yes | Yes | No | Yes | Yes |
| deVos et al. 2009^[14]^ | No | No | Yes | Yes | No | Yes | Yes |
| Lee et al. 2009^[12]^ | Yes | No | Yes | Yes | Yes | Yes | Yes |
| Grutzmann et al. 2009^[15]^ | Yes | No | Yes | Yes | Yes | Yes | Yes |
| Lofton-Day et al. 2008^[13]^ | No | No | Yes | Yes | No | Yes | Yes |
| Wang et al. 2008^[56]^ | No | No | Yes | Yes | No | Yes | Yes |
| Zhang et al. 2006^[57]^ | No | No | Yes | Yes | No | Yes | Yes |
| Lu et al. 2006^[21]^ | No | No | Yes | Yes | No | Yes | Yes |
| Ebert et al. 2006^[26]^ | No | No | Yes | Yes | No | Yes | Yes |
| Leung et al. 2005^[58]^ | No | No | Yes | Yes | No | Yes | Yes |
| Zou 2002^[24]^ | No | No | Yes | Yes | No | Yes | Yes |

Quality was assessed with the Quality Assessment of Diagnostic Accuracy Studies-2 tool.
